# Advances in Unmanned Aerial Vehicle-Based Sensing and Imaging

**DOI:** 10.3390/s24248094

**Published:** 2024-12-19

**Authors:** Marios Antonakakis, Michail Zervakis

**Affiliations:** Electrical and Computer Engineering, Technical University of Crete, Aktotiri Campus, GR-73100 Chania, Greece; mzervakis@tuc.gr

Unmanned Aerial Vehicle (UAV)-based sensing and imaging represent a cutting-edge approach to addressing the critical need for the real-time, accurate, and automated data collection and analysis of physical events on Earth’s surface. This technology has rapidly evolved, compiling recent trends in UAV-equipped sensors and analytics for studying various phenomena, particularly those involving flora and fauna, terrain development, and species movement, even through dense foliage. The versatility of UAV platforms, equipped with an array of sensors including optical, acoustic, hyperspectral, infrared, RADAR/SAR, and LiDAR, has transformed how we capture and interpret data, pushing the boundaries of machine learning, computer vision, and data processing tasks. Recent technological breakthroughs have solidified UAV-based imaging as a preferred method, providing advantages in efficiency, accessibility, and cost-effectiveness compared to orbital and traditional aerial methods.

This Special Issue aims to collect and categorize innovative scientific contributions that explore advanced sensing technologies, multisensory systems, and cutting-edge algorithmic developments. With the surge in the development of sensors that are capable of real-time object detection on embedded systems and the integration of multi-sensing devices, UAVs have become indispensable tools for acquiring detailed data on the Earth’s surface. These advancements are further powered by the growing capabilities of deep neural networks, which have enabled robust and intelligent methods for mapping and analyzing the environment. However, the challenges of automating, accelerating, and ensuring the accuracy of remote sensing data remain at the forefront of research and development.

The collected articles in this Special Issue reflect the diverse applications and transformative potential of UAV technology. For instance, advancements in construction safety have been achieved through UAV systems equipped with Faster R-CNN models, enabling the real-time detection of workers’ compliance with safety protocols and significantly reducing injuries and fatalities. In the realm of 3D mapping, the FaSS-MVS system presents a surface-aware multi-view stereo approach that facilitates efficient and accurate depth and normal map estimation, outperforming traditional methods in speed and scalability.

Thermal imaging is another area where UAVs have shown significant progress. The development of ThermoSwitcher, a thermal image format conversion system, has enhanced preprocessing efficiency, enabling refined surface microthermal environment analysis. Archeological applications also benefit from UAV-borne magnetic gradiometry, which provides high-resolution data for detecting buried structures while overcoming noise and instability challenges.

Wildfire detection has been revolutionized by the integration of enhanced YOLOX networks, improving small-target detection accuracy and efficiency in complex fire environments. Similarly, UAVs are being used for real-time power line monitoring by integrating RGB and thermal imaging, reducing operational costs while enhancing fault detection. Other applications include advanced object detection algorithms, acoustic surveillance, and bathymetric modeling using neural networks for shallow water areas, showcasing the versatility and reliability of UAV platforms across various domains.

As UAV technology continues to evolve, this Special Issue highlights its critical role in advancing sensing and imaging research. By integrating state-of-the-art sensors, deep learning algorithms, and innovative methodologies, UAVs have become essential tools for addressing complex challenges in environmental monitoring, disaster response, infrastructure inspection, and beyond. These contributions not only reflect the technological advancements but also underline the practicality and human applicability of UAV-based systems, paving the way for further innovations in remote sensing and imaging.

